# Quality of life assessment in women with breast cancer: benefits, acceptability and utilization

**DOI:** 10.1186/1477-7525-5-24

**Published:** 2007-05-02

**Authors:** Sheila Perry, Theresa L Kowalski, Chih-Hung Chang

**Affiliations:** 1 Buehler Center on Aging, Health & Society, Northwestern University Feinberg School of Medicine. 750 N. Lake Shore Dr., Suite 601, Chicago, Illinois 60611, USA; 2 Division of General Internal Medicine, Department of Medicine, Northwestern University Feinberg School of Medicine, Chicago, Illinois, USA

## Abstract

In 2006, breast cancer was the third leading cause of death in American women; however, more women survive breast cancer than any other type of cancer. As the disease progresses, it is important to know how one's health-related quality of life (QOL) is affected for those who receive treatment, those who survive, and those who remain disease-free. The purpose of this study was to summarize the benefits, challenges, and barriers of QOL measurement for female breast cancer patients. A PubMed literature search was conducted using the terms "quality of life" and "breast cancer." The search was then refined with terms related to QOL assessment instruments. The research team reviewed over 100 of the 2,090 articles identified.

From the results, a detailed outline of QOL instruments is presented, and the effectiveness of QOL instruments is discussed. In the current literature review, both generic and breast cancer specific QOL instruments, examining computerized and paper-and-pencil versions, are explained as well as the advantages, acceptability, and problems of these assessments. Potential barriers to implementation are also discussed. The implementation of QOL assessment tools in breast cancer clinical practice is discussed, with evidence detailing how such tools would benefit patients.

## Background

### The burden of breast cancer

Breast cancer is costly, both in human and economic terms. In 2006, breast cancer was the third leading cause of death in American women after heart disease and lung cancer, accounting for more than 41,000 deaths [1]. Breast cancer also accounted for 31% of all cancers in females and 15% of all cancer-related deaths for women in the U.S. in 2006 [2]. Even though heart disease is the leading cause of death in females, breast cancer is the disease many women fear the most [3]. It is estimated that one in eight American women will be diagnosed with breast cancer at some point in their lives [4]. The number of women with breast cancer is increasing annually. Each year, over 1.1 million females worldwide are diagnosed with breast cancer and 410,000 women die from the disease[5]. According to the American Cancer Society, breast cancer was the number one newly diagnosed disease for women in 2005 and accounted for 31% of all newly diagnosed cases in 2004. More than 9.5 million women were learning to cope with a progressive disease, going through curative treatment, or living cancer-free after treatment in 2004 [6].

Breast cancer is an economic burden, with its cost of illness being comprised of direct cost, morbidity cost, and mortality cost. Over extended periods, cancer expenditures are increasing at a similar rate to overall health costs [7]. According to the National Institutes of Health, breast cancer was estimated to cost $209.9 billion a year in 2005, of which $118.4 billion was due to mortality cost (lost productivity by the death of patients), $74.0 billion was due to direct medical costs (money spent for healthcare), and $17.5 billion was due to morbidity cost (the cost of lost productivity due to illness) [8]. Longitudinal data on cancer costs exist on the SEER-Medicare linked database, a resource for policy makers and analysts, which helps to represent the costs of cancer based on its prevalence and incidence [7].

### The effects of breast cancer on patients

Breast cancer patients experience physical symptoms and psychosocial distress that adversely affect their quality of life (QOL). QOL generally consists of a number of domains including physical functioning, psychological well-being (such as levels of anxiety and depression), and social support. Their breast cancer experiences vary, but could include the following phases: diagnosis, primary treatment, genetic risk and its psychological management, special issues related to non-invasive breast cancer, recurrence, completing treatment and re-entry to normal living, survivorship, and palliation for advanced cancer [9]. Chemotherapy, for example, is one form of treatment that can cause physical and psychological problems that adversely affect patient QOL [10]. Other effects of cancer include anger, grief, suffering, and pain [11]. While adapting to cancer, many patients may have questions about their illness, but are apprehensive about speaking to their physician [11].

Psychosocial problems compound the hardships of physical symptoms and affect the QOL of breast cancer patients. The psychosocial distress that patients upon diagnosis feel can affect their treatment because these symptoms can be overwhelming. Many women who are newly diagnosed with breast cancer might feel sad, anxious, shocked, and scared. Psychological treatments could help patients come to terms with their emotions and treat mental illnesses they may develop, including depression, panic disorders, and anxiety disorders [9].

Sharpe et al. reported that another important aspect of living with cancer is the process a person uses to adjust to his or her illness due to acclimation of the illness. Specifically, this occurs when a person shifts his or her priorities and expectations during assessments to be in line with his or her differing circumstances [12]. They found that patients who were recently diagnosed with metastatic cancer reported more areas of their life as being important to their QOL each time they were asked. In particular, almost half (47%) of the participants shifted the areas they described as being the most important between the initial questioning and the second questioning three months later. Forty-three percent shifted their responses between the second and third questioning (six months after the initial questioning) [12].

### Breast cancer treatments and quality of life

Objective tumor response and survival traditionally have been used to assess cancer treatment outcomes. Two major changes in cancer medicine have occurred over the past decade. The first is recognition that the patient's well-being is important to cancer treatment. Another is the use of QOL and psychosocial questionnaires to assess their well-being. Since the time of Hippocrates, QOL has been an implied medical outcome [13,14]. In 1948, Karnofsky et al. [15] reported the first effort of physicians to assess systematically the effect of cancer treatments on patients' QOL, not just on their quantity of life. QOL instruments currently are being used in clinical trials to predict survival, response to treatment, and to screen for psychological morbidity [16].

Understanding the effect of breast cancer treatment on a patient's QOL has been a central clinical and research question. For the past quarter century, psychosocial and emotional concerns have been addressed in intervention research of women with breast cancer. Findings by Ganz and Goodwin revealed that with the number of survivors growing in recent years, breast cancer patients have been assessed with multiple QOL instruments in order to compare the effects of breast cancer and its treatments to those of people with other chronic illnesses as well as to healthy women [17].

One important issue regarding the implementation of QOL assessment is the use and utilization of validated instruments within a clinical setting, not specifically for research purposes[18]. QOL consists of several domains including physical functioning, psychological well-being (such as levels of anxiety and depression), and social support. Donaldson [18] states that patients report both positive and negative effects of their diagnosis and treatment. QOL assessments can be used in diagnosis, predicting prognosis, assessment, patient monitoring, clinical decision-making, communication, and treatment. Other uses include designing system intervention, allocating resources and research efforts, training health care personnel, and reducing costs [18,19]. Which specific instrument is chosen is based on: the purpose of the assessment, the patient population targeted, and the timing and frequency of the administration of a given instrument [18].

Instrumental in reviewing breast cancer outcomes, Mandelblatt and colleagues (2004) [20] conducted a meta-analysis utilizing 230 articles from 1990 to 2000. Primary conclusions drawn from this study include the majority of articles utilized health-related QOL outcomes, no single instrument was utilized in more than 10% of all the articles, most articles had a primarily white population and did not focus on elderly breast cancer patients, and methodological flaws were prevalent throughout the studies analyzed.

### The importance of meeting the needs of breast cancer patients

Studies by Ganz and Goodwin found that when assessing the QOL of women with breast cancer, their stage of disease should be taken into account and include: women with non-invasive breast cancer, women who are newly diagnosed with early stage breast cancer or advanced local breast cancer, women surviving disease-free beyond the first course of primary treatment, survivors of five years or more, women with a first recurrence of breast cancer after a disease-free interval, and women living and dying with advanced metastatic disease [17].

Some cancer patients may be unwilling to reveal their concerns about their disease and treatment [21], and may be even more unwilling to raise psychological problems they may develop throughout the course of their disease [22]. While patients want their health care providers to inquire about their daily functioning and well-being, health care providers may seldom do so [23-25]. However, if physicians were not concerned about their patients' outcomes, there would be no reason for follow-up visits [18]. Oncologists, nurses, and psychosocial staff agree that QOL is an important variable to consider in cancer treatment [26-28]. However, patients and physicians frequently have different priorities regarding treatment and the effect that the illness will have on a patient's life and possible outcomes. Employing the patients' views into the decision process would not only empower patients, but also could improve satisfaction and compliance with treatment. Sprangers and Schwarz stated that patient outcomes could be improved further by utilizing QOL assessments to detect and treat functional and psychological issues that have not been brought previously to the forefront. People whose expectations are met in the areas they consider as most important are those who report a good QOL [29].

### The purpose of this review

The primary purpose of this paper is to expand the work of Mandelblatt and colleagues [20] into recent years, focusing on QOL assessments and new research possibilities utilizing computerized QOL assessments. This review aims to add to the scientific literature by revealing the answers to five significant questions about the role of QOL assessments for breast cancer patients and suggesting future directions for the field. First, how would the implementation of QOL assessments into clinical practice benefit the overall QOL of patients? Second, to what degree have healthcare providers found QOL assessments acceptable to utilize in clinical practice? Third, how can QOL data be utilized to benefit patients? Fourth, how can barriers to implementation be overcome? Fifth, how can technology improve the accuracy of these assessments and facilitate their implementation into clinical practice?

## Methods

A systematic review was conducted by searching the PubMed database, limiting the research to the years 1995–2005, and using the following keywords: quality of life and breast cancer (in all of the searches) in conjunction with computer, software, touch-screen, program, assessment, questionnaire, instrument, and patient-reported outcomes separately within each search. Over 2,090 articles were found meeting the criteria. The research team reviewed over 100 of the most relevant articles for inclusion in this report.

Inclusion criteria included articles published in a peer-reviewed journal, specifically utilizing QOL assessments with chronically ill patients (particularly female breast cancer patients), articles examining the use of QOL assessments in randomized trials, studies involving both paper and pencil versions of the instruments as well as computerized versions of the assessments, and literature reviews concerning QOL of breast cancer patients. Exclusion criteria included comments/letters and papers published in a language other than English.

The second stage of the research involved searching the PubMed database between the years 1989–2006 using the following keywords: quality of life and breast cancer (in all of the searches), in addition to advantages, acceptability, patient acceptability, problems, barriers, and implementation. The 20 most relevant articles from this search were included in the study.

## Results

Because of the complexity of breast cancer and the diverse nature of its patient population, no one instrument is both comprehensive and sensitive enough to report clinically meaningful changes in all outcomes across all phases of care and has satisfactory respondent or provider burden. However, based on Mandelblatt's et al. [20] comprehensive meta-analysis of breast cancer outcomes literature, they believe it is possible to develop a "core" set of questions to measure breast cancer outcomes. After reviewing the articles retrieved in the PubMed search, 21 QOL instruments were identified as being the most used assessments within the breast cancer population. Each instrument is briefly described below and a summary of these instruments can be found in Appendix A (see Additional file 1).

### Description of QOL instruments

#### Beck Depression Inventory (BDI) [30]

The BDI is a 21-item self-administered scale designed to measure depression. It has one domain, which is depression.

#### The Breast Cancer Chemotherapy Questionnaire (BCQ) [31]

The BCQ is a 30-item interviewer-administered questionnaire developed to measure outcomes of women with stage II breast cancer receiving adjuvant chemotherapy. It is comprised of seven domains (consequences of hair loss, positive well-being, physical symptoms, trouble and inconvenience, fatigue, emotional dysfunction, and nausea) [31].

#### Breast Cancer Prevention Trial Symptom Checklist (BCPT) [32,33]

The BCPT is a 43-item self-administered questionnaire designed to examine the physical and psychological symptoms associated with menopause and Tamoxifen usage. This questionnaire is comprised of eight symptoms (hot flashes, nausea, bladder control, vaginal problems, musculoskeletal pain, cognitive problems, weight problems, and arm problems).

#### Cancer Needs Questionnaire – Short Form (CNQ-SF) [34]

The CNQ-SF is a 32-item self-administered questionnaire developed to assess cancer patients' needs. It is comprised of five domains (psychological, health information, physical and daily living, patient care and support, and interpersonal communication) [35].

#### Cancer Rehabilitation Evaluation System – Short Form (CARES-SF) [36]

The CARES-SF is a 59-item self-administered questionnaire developed to assess patients' cancer-related problems. The assessment is comprised of six domains (physical, psychosocial, medical interaction, martial, sexual, and global) and is scaled on a five point Likert scale ranging from 0 (Not at all) to 4 (Very much) [37].

#### Center for Epidemiologic Studies Depression Scale-10 (CES-D) [38]

The CES-D-10 is a short 10-item self-administered scale designed to measure depression[39]. The 20-item CES-D is one of the most common screening tests for depression.

#### Edmonton Symptom Assessment System (ESAS) [40]

The ESAS is a 9-item self-administered questionnaire designed to measure a variety of symptoms. This questionnaire is comprised of nine domains (pain, tiredness, nausea, depression, anxiety, drowsiness, appetite, well-being, and shortness of breath) and is scaled using a visual analog scale[41].

#### European Organization for Research and Treatment of Cancer QOL Cancer Specific Version (EORTC QLQ-C30)[42]

The EORTC QLQ-C30 is a 30-item self-administered cancer specific questionnaire designed to measure QOL in the cancer population. The assessment is comprised of nine domains (physical, role, cognitive, emotional, social, fatigue, pain, nausea and vomiting [43].

#### European Organization for Research and Treatment of Cancer QOL Breast Cancer Specific Version (EORTC QLQ-BR23) [44]

The EORTC QLQ-BR23 is a 23-item self-administered breast cancer specific questionnaire, usually administered with the EORTC QLQ-C30, designed to measure QOL in the breast cancer population at various stages and with patients with differing treatment modalities. The assessment is comprised of five domains (body image, sexuality, arm symptoms, breast symptoms, and systemic therapy side effects) [44].

#### Functional Assessment of Cancer Therapy – Breast Symptom Index (FACT-B – FBSI) (44 items) [45,46]

The FACT-B – FBSI is a 44-item self-administered questionnaire specific to breast cancer patients. The assessment is comprised of six domains (physical well-being, social/family well-being, relationship with doctor, emotional well-being, functional well-being, and additional concerns) [45].

#### Functional Assessment of Cancer Therapy – Endocrine Symptom Subscale (FACT-ES) [47]

The FACT-ES is an 18-item self-administered questionnaire, usually administered with the FACT-B, focusing on endocrine concerns experienced during breast cancer treatment [47].

#### Functional Living Index – Cancer (FLIC) [48]

The FLIC is a 22-item self-administered questionnaire designed to assess the effect cancer treatment and symptoms have on functional ability in all areas of life. This questionnaire is comprised of five domains (physical functioning, mental functioning, social functioning, general health/well-being, and gastrointestinal symptoms) [48].

#### Geriatric Depression Scale – Short Form (GDS-SF)[49]

The GDS-SF is a 15-item self-administered questionnaire designed to assess depression in the elderly. This instrument is comprised of four domains (positive mood; sad mood; boredom, memory problems, and energy level; and staying at home) [50].

#### Hospital Anxiety and Depression Scale (HADS) [51]

The HADS is a 14-item self-administered questionnaire developed to measure anxiety and depression. The questionnaire is comprised of two domains (anxiety and depression) [52].

#### Life Satisfaction Questionnaire (LSQ) [53]

The LSQ is a 32-item self-administered questionnaire developed to measure one's general sense of satisfaction with life as it relates to school, relationships, leisure time, religious practices, and overall health specifically for women with breast cancer. This questionnaire is comprised of six domains (quality of family relation, physical symptoms, socioeconomic situation, quality of daily activities, sickness impact, and quality of close friend relation) [53].

#### Medical Outcome Study's 36-Item Short Form Health Survey (SF-36) [54]

The SF-36 is a 36-item self-administered questionnaire developed to assess health-related QOL. It is comprised of eight domains (physical functioning, role limitations due to physical health, role limitations due to emotional problems, energy/fatigue, emotional well-being, social functioning, bodily pain, and general health) [55].

#### Rotterdam Symptoms Checklist (RSCL) – Modified[56]

The RSCL-Modified is a 28-item self-administered questionnaire developed to assess symptom-related distress among cancer patients. This instrument is comprised of two domains (physical distress and miscellaneous variables) [56].

#### Satisfaction with Life Domains Scale for Breast Cancer (SLDS-BC)[57]

The SLDS-BC is a 32-item self-administered questionnaire comprised of five domains (social functioning, appearance, physical functioning, communication with medical providers, and spirituality) [57].

#### Spitzer Quality of Life Index (QL-Index)[58]

The QL-Index is a 5-item interviewer-administered or self-administered questionnaire designed to assess health outcomes of those with cancer and other chronic diseases. The questionnaire is comprised of five domains (activity, daily living, health, support, and outlook) [58].

#### World Health Organization Quality of Life – Brief Version (WHOQOL-BREF) [59]

The WHOQOL-BREF is a 26-item self-administered assessment designed to examine domain level profiles assessing quality of life. This assessment is comprised of four domains (physical health, psychological, social relationships, and environment) [60].

#### Zung Self-Rating Depression Scale (SDS) [61]

The Zung SDS is a 20-item self-administered scale designed to measure depression [62].

### Benefits of QOL assessments

#### Overall benefits of QOL assessments

The overall benefits of QOL assessments include their use in preventive intervention and their potential to inform clinicians about the patient's illness as well as how certain treatments may affect the QOL of that patient. Jacobsen et al. report that an understanding of QOL also improves communication between physicians and patients [63]. Evaluation of the advantages of QOL assessments has led the National Institutes of Health and the National Cancer Institute to conclude that QOL measures should be incorporated into research studies when possible.

#### Benefits of QOL assessment in clinical practice

Magruder-Habib et al. [64] and Rubenstein et al. [65] reported significantly more changes to treatment in the intervention group, participants who were assessed using a QOL instrument, as compared to the control group, who were not assessed. In one study, physicians took more action on the patient-reported categories listed in the chart of the experimental group as compared to the control group (73% and 68.5%, respectively) [64]. Gold and Baraff [66] and Mazonson et al. [67] reported significantly more referral rates to other professionals in the intervention group as compared to the control group. One study reported that physicians found the questionnaires to be more useful for those patients who had a worse initial functioning score [24].

In a study by Detmar et al. [68], almost 97% of the patients stated that the QOL profile provided an accurate summary of their functioning and well-being. Fifty-seven percent reported their physicians used the profile explicitly during their visits, 79% believed the profile enhanced physician awareness of their health problems, and 87% thought it would be useful to introduce a QOL assessment as a standard part of the outpatient clinical experience. With regard to the length of time for an office visit, this study did not find statistical significance in the length of office visit; however, the control group visits took longer than the intervention group (20.4 minutes vs. 19.8 minutes, respectively) [68].

#### Role of QOL assessment in facilitating communication

A study conducted by Mazonson et al. [67] reported an increase in patient-physician communication in the intervention group as compared to the control group. Wagner et al. [24] found a moderate percentage (67%) of patients reported positive attitudes about completion of the assessment as well as sharing their feelings and physical abilities with their physician. Furthermore, the study reported that more patients wanted physicians to inquire about their feelings (80%) as opposed to their physical abilities (71%). Street et al. [69] reported that most patients wanted physicians to inquire about their vitality, physical functioning, and role limitations due to physical issues, and only half of the patients felt that these problems were currently being discussed. Other studies reported that at least a moderate percentage of physicians said that they had made changes within their practice since the implementation of a QOL instrument [70-72].

Studies have reported that QOL issues in intervention groups were discussed significantly more frequently when QOL assessments, specifically the EORTC-C30, were used compared to control groups (no QOL assessment used) [68,73]. Taenzer et al. revealed that patients reported 49% of the QOL items they identified were addressed by their physician during their clinical visit [73]. In Detmar et al. [68], 10 of the 12 QOL issues were discussed more frequently in the intervention group; however, only three issues reached statistical significance: social functioning, fatigue, and dyspnea. Intervention group physicians were able to identify a larger percentage of patients with moderate to severe QOL issues, such as problems with daily activities, feelings, social activities, pain, and fatigue, compared to the control group's physicians, who only could identify problems in daily activities and pain. The most noteworthy increase was the discussion of less observable issues, such as social functioning, or those that are more diffuse and long-term in nature, such as fatigue, that are usually left unaddressed by physicians [68].

In the same study by Detmar et al. [68], patients in the intervention group received significantly more counseling from their physicians on how to manage their health problems than did the control group. In examining patient satisfaction, the only statistically significant difference between the two groups was shown for the amount of emotional support received, with the intervention group receiving more than the control group. Overall, patients in the intervention group exhibited a statistically significant improvement over time in regards to mental health and role functioning. All of the physicians stated that the profiles offered a useful overall representation of their patient's health-related experiences and indicated that it also assisted with communication, particularly in regards to psychosocial topics and "unexpected" symptoms such as sleep disturbances [68].

#### Role of QOL assessment in clinical decision-making

Bendsten et al. reported that physicians have reported that a holistic approach, including QOL, is necessary when treating patients [74]. Greenhalgh and Meadows, Di Maio and Perrone, and Le et al. have found that one important benefit of QOL assessment is to encourage shared decision making and to facilitate communication between physicians and patients[25,75,76] by providing feedback to the patients regarding their progress, goals, and expectations [18]. According to Donaldson as well as Deyo and Carter, QOL measures can also be used for monitoring disease progression (i.e., survival) or response to treatment (i.e., toxicity, side effects, and adverse effects) [18,77]. Other potential benefits of QOL measurement include detection of treatable issues that are normally overlooked during patient care such as education about the disease and nutritional counseling as well as obtaining a baseline upon entering care or therapy. QOL measurement could potentially be helpful in monitoring a reduction of functional capacity and in identifying other physical and emotional problems. Donaldson as well as Bendsten et al. and Le et al. have found that timely feedback can result in a reduction in anxiety, thus preventing fewer visits and calls with improved satisfaction with care [18,74,76]. If patients were to take on the role of more self-management with the use of QOL assessment's implementation, it could lead to more efficient use of resources, and thereby reduce costs [18].

Detmar et al. [68] (2002) discovered that even though all of the physicians reported they would like to continue using QOL assessments in their daily practice, several suggested the need for more tailored assessments to specific patient groups, such as the site of pain and use of pain medication for patients with bone metastases [68]. Another study by Middeke et al. found that all the participating physicians reported the QOL profiles were easy to understand and clinically relevant, and more than half of the physicians indicated the profiles had provided them with additional information that led to a more complete diagnosis [78].

Treatment plans can also be altered or contingent upon an individual's QOL. Di Maio and Perrone [75] found that elderly cancer patients prefer treatments that potentially improve their QOL over that of their survival; only 22% chose chemotherapy if a hypothesized survival benefit of three months was given, and 68% chose chemotherapy if it would substantially reduce symptoms, even if no significant increase in survival was expected.

#### Benefits of non-visit based assessment platforms

A study by Donaldson revealed that the Internet, computers, telephones, and hand held devices now allow QOL assessment to become non-visit based. These platforms provide a highly efficient, timely, and individualized approach to QOL assessment, and provide better privacy and confidentiality.

By allowing patients to complete password-protected QOL assessments whenever they are away from doctors' offices and are experiencing symptoms, patients may submit their reports at any time of the day. PRO reports submitted during the day can be reviewed immediately by physicians, and those submitted after office hours would be viewed the next morning. By evaluating PRO reports, physicians may triage patients to determine which cases are most urgent and to decide on appropriate treatment customized for each patient. Triage is usually performed by using a threshold score, with people above the determined score to be given more urgent care. These methods also allow for ongoing monitoring of patient treatment and management to evaluate their effectiveness [79].

### Acceptability of QOL assessments

#### Overview

Healthcare providers and patients alike find QOL assessments acceptable in helping breast cancer patients to overcome areas of QOL that have been affected by the disease. Several studies have demonstrated the reasons why the implementation of QOL assessments is found acceptable by physicians.

#### Healthcare provider acceptability

Finlay and Dunlap as well as Bezjak et al. report that healthcare providers agree that QOL assessments are valuable in clinical practice [26,28]. Bezjak et al[28] (2001) conducted a survey of 271 Eastern Cooperative Oncology Group (ECOG) physicians and found that 94% of respondents *disagreed *with the statement "I do *not *plan to incorporate QOL data in my practice." In addition, 75% *disagreed *with the statement "I would only be willing to use formal QOL assessment if required to do so by my institution or regulatory body." Eighty-two percent indicated it was likely that they would increase their use of QOL in the care of future patients [28].

Several studies, including those done by Wagner et al., Wasson et al., and Moore et al., have reported at least a moderate percentage of physicians had positive attitudes about the implementation and feasibility of QOL assessments within routine practice [24,70,80]. Moore et al. [80] and Rubenstein et al. [71] showed a large percentage of internists and family physicians reported the QOL information as accurate and would use the instrument with other patients in the future. Other studies examined the physician's perception of the efficacy of the measures in routine practice. All of them found at least a moderate percentage of physicians who showed positive attitudes [24,70-72,80,81].

Another study by Taenzer et al. found an overwhelmingly positive response from oncology nurses regarding the usefulness of a computer-administered questionnaire's generated report, with 91% stating the report indicated appropriate concerns, 91% stating it guided the clinical interaction, 82% stating it promoted communication, and 74% stating the report indicated areas of need for the patients [82]. Velikova reported that when assessing the oncologists' checklists, it was found the clinicians thought the health-related QOL information was very useful in 43% of the visits, somewhat useful in 28% of the visits, a little useful in 21% of them, and not useful in 9% of the visits [83].

#### Patient acceptability

Stiggelbout et al. [84] found that it was feasible to use questionnaires to evaluate patients' feelings regarding quality of life versus quantity of life. Apolone et al. [85] examined the acceptability of QOL questionnaires among patients and found the response rate was 64% with few missing items on the questionnaires. These results provide an example of the acceptability of QOL questionnaires for patients, and the EORTC QLQ-C30 in particular for this study [85].

To be acceptable, questionnaires must be sensitive to the needs of patients. Cancer patients found assessments that asked questions in the form of words rather than only numbers and had fewer answer options to be acceptable. Additionally, studies by Pijls-Johannesma et al. have revealed that questionnaires that did not ask questions that were overly personal, sensitive, or irrelevant were favored by respondents [86].

### Problems with QOL assessments and barriers to implementation

#### Overview

Morris et al. [87] found that although 80% of healthcare professionals believed that information obtained from QOL assessments is valuable, fewer than 50% of them implemented QOL assessments in their practice. The researchers attributed the problems to an unwillingness of physicians to use the assessments because they are considered unnecessary, the belief of some practitioners that they do not have appropriate instruments, and logistical problems such as limited resources and time [87].

#### Problems due to characteristics of QOL assessments

Even though QOL information is clinically valuable, physicians often view it as providing "soft" data that does not permit "hard" measurement such as that obtained in the laboratory. Barriers to implementation can also arise due to the types of questions asked. Questions that are too personal, sensitive, or irrelevant are more likely to be omitted by respondents. For example, some questionnaires ask respondents about their careers. Pijls-Johannesma et al. report that such questions are irrelevant in the elderly population, as many respondents are retired [86].

#### Problems with implementation related to patient population

Potential problems with QOL assessments can occur prior to their implementation in clinical practice. Few studies have yet assessed QOL on long-term survivors (greater than 5 years after diagnosis), as most assess patients one year after diagnosis. It is necessary to include long-term survivors who are willing to participate in the study so that their views can be fully integrated into the initial instrument development.

Particularly in the elderly, QOL assessments can be difficult to incorporate into a clinical practice because there are a higher rate of illiteracy, worse compliance with questionnaires, cognitive disorders, comorbidities, and non-validated instruments within this population. Di Maio and Perrone [75] found a decrease in compliance in the elderly, as only 21.5% of the elderly patients completed the QOL questionnaire administered after six months, whereas 37.2% of younger patients completed the questionnaire. As mentioned above, Detmar et al. [68] did not find any significant difference in the length of time between the control visit and the intervention visit.

#### Problems with implementation related to their use by healthcare professionals

Findings by Deyo and Carter reveal that physicians are less familiar with QOL assessments than with imaging or physiological tests and often are unfamiliar with how to interpret the results or respond to them [77]. Limited time is often mentioned as one of the problems associated with utilizing a QOL assessment in daily practice; however, one study by Maguire et al. found that it only took an average of 60 seconds longer for the physician to extract all of the patient's current problems [88].

#### Problems with implementation due to logistical and resource constraints

Gotay et al. state that other barriers to implementation of QOL assessments include staff and financial limitations with respect to the amount needed to produce high-quality data, quality control, monitoring procedures, data collection, and submission [89]. Some researchers believe that interviews or semi-structured interviews conducted by professionals should be used instead of self-assessments to minimize the amount of missing data [11].

As reported by Velikova et al. and Allenby et al., QOL measures traditionally are collected by printed self-report questionnaires and entered into a database manually [16,90], which, according to McDonald and Barnett, is still the most commonly used method [91,92]. A study by Le et al. revealed that this method, however, is fraught with high labor costs, poor turnaround time, and errors [76]. Fairly recently, optical mark-recognition systems have been developed to enhance the transfer of the data into databases. This form of input can process a large amount of data and is practical for mailed questionnaires. Nevertheless, this system has problems, such as requiring special forms, recognizing multiple answers, missing data, and examining the database for errors. Velikova et al. and Allenby et al. report that more recently, electronic means of administering QOL assessments have been introduced which combat some of the problems associated with the printed forms and optically read systems. Results are compiled automatically in the database and are available in real time for clinical use [16,90].

### Computerized assessment

#### Overview

Computerized QOL assessments have several advantages. They provide more accurate results, and as such, represent a picture of patients' QOL more clearly. Additionally, the use of technology to facilitate the implementation of QOL assessments increases efficiency and allows utilization by a wider population.

According to a study by Buxton et al., a touch-screen computerized system has advantages over paper-and-pencil QOL questionnaires, which include the ability to increase the font size for those with visual difficulties, reduce the number of missed items, decrease the need for paper, analyze the patient's information immediately, and easily store and retrieve the data [93]. Allenby et al. report that electronic completion of QOL questionnaires also can reduce the potential of errors with after-survey data input and can reduce the length of time it takes to administer the questionnaires [90]. Taenzer et al. [82] found patients took an average of 8.6 minutes to complete an electronic version of the EORTC QLQ-C30 questionnaire as opposed to 11 minutes to complete its paper version. In another study by Velikova et al. examining the difference between the electronic version of the EORTC QLQ-C30 and its paper version, patients took less time to complete the touch-screen version (8.3 minutes) as opposed to the paper version (9.6 minutes) [16]. Bliven et al. state that the Internet allows for the collection of patient-reported data at multiple locations, at frequent time intervals, and at little cost [94]. It is also reported by Le et al. that touch screens or pen-based computers are appealing because they are portable and do not require the respondents to use a keyboard or a mouse [76].

In a study by Velikova et al. comparing the touch-screen versions and paper versions of the EORTC QLQ-C30 and the HADS, the quality of the data extracted from a touch-screen version was found to be excellent, with no missing or problematic responses, mainly because the patient could not progress through the questionnaire without answering each question. In contrast, 1,032 errors were reported from the paper version; 357 being patient related errors (such as missing responses, multiple answers, or changed answers) and 725 being scanner related errors (such as not recognizing the responses). More than 92% of the optically read questionnaires had scanning, verification, or database errors, which caused significant additional work for the research staff who had to check and verify the quality of the database after each scanning of the paper questionnaires [16]. The same study found that 52% of the patients surveyed preferred the touch-screen computer, compared to 24% preferring the paper version. Overall, 76% of all patients found the computer version to be acceptable (either preferring it specifically or finding it as acceptable as the paper version). Administration preference was not found to be related to age, gender, order of presentation, or time for completion [16].

In one study by Allenby et al., patients reported the touch-screen system to be very easy to use in regards to their ability to read the screen (95% of the patients), entering of an identification number (76%), following the instructions (74%), and choosing the appropriate answer (71%) [93]. In another study, 99% of patients found the touch screen easy to use, 98% stated the instructions were easy to follow, 98% stated the questions were easy to read on the screen, and 99% stated the time it took to complete the survey was acceptable [90].

Another study by Taenzer et al. found 41% of women liked computer-administered questionnaires, 45% were neutral, and 14% said they did not like such questionnaires. If participants had prior experience with the program, their attitudes towards the computer-administered questionnaire changed, with 80% stating they liked the method, 11% stating they were neutral, and only 8% stating they did not like the method [82]. Similarly, Newell et al. reported that 89% of oncology patients stated they would be willing to fill out a subsequent touch-screen questionnaire, with 73% of those patients being over the age of 50 and 59% having never used a computer before the assessment [95]. Bliven et al. [94] found that in spite of nearly half of the respondents stating they had no prior computer experience, computer literacy was not significantly associated with the respondent's ability to complete successfully the computerized questionnaire.

A study by Velikova et al. that examined the use of computer technology in QOL assessments found that primary outcomes on the FACT-G [45] showed a significant improvement in the intervention group, who completed the EORTC QLQ-C30 [42] and the HADS [51] on a touch-screen computer in the clinic and received feedback from the physician, as compared to the control group, who did not complete questionnaires before the visit. With further analyses, a significant improvement was also detected between the attention-control group, who completed the questionnaires but did not receive any feedback from the physician and the control group. In examining emotional well-being, the intervention group showed a higher score than the control. No difference was found, however, between the attention-control and control groups. Those patients who decided not to complete the study had a significantly lower FACT-G score at baseline than patients who finished the study. A sizeable percentage of patients in the intervention group had significant improvement in QOL after only three visits. The average number of visits needed for one person to benefit was 4.2 [83].

In examining the transcripts of the visits in the above-mentioned study, it was found that the intervention group had a higher number of EORTC QLQ-C30 symptoms being discussed than the control group. More discussion of chronic symptoms, such as difficulty sleeping, lack of appetite, and fatigue were observed without extending the visits. When QOL data were discussed with patients during their third visit, the mean change of the FACT-G score was higher than when QOL data were not mentioned [83].

#### Problems with computerized assessment

Velikova et al. found that patients had difficulty with the handheld computers because of the small screens as well as the software design, which prevented the patients from changing their answers. The handheld computers also had technical difficulties resulting in unreliable downloaded data and administrative problems in ensuring the security of the portable computers and the data contained within them [16]. Entering the demographic information on the touch-screen computer was difficult and time consuming because if patients made a mistake, they would have to delete their responses and start again.

Another study by Buxton et al. found 23% of patients had problems using the touch-screen because of its lack of appropriate responsiveness to touch due to the wearing-down of equipment towards the end of the study [93]. It would be of importance to include potential users of QOL assessments from the beginning to determine confounding issues and correct them before implementation of the system.

## Discussion

### Overview

The overarching purpose of this review has been to add to the scientific literature by revealing how the implementation of QOL assessments into clinical practice would benefit the overall QOL of patients, by evaluating the acceptability of QOL assessments among healthcare professionals, by addressing how QOL assessments can be utilized, by suggesting solutions to the challenges of implementation, and by revealing how technology can improve the accuracy of QOL assessments and facilitate their implementation into oncology practices.

### The overall acceptability and benefits of QOL assessments for breast cancer patients

QOL assessments have found acceptability among physicians, nurses, and psychosocial staff for several reasons. QOL assessments benefit breast cancer patients because they provide insights into life domains affected by breast cancer that are usually unaddressed, including a patient's mental health, emotional well-being, family and social relations, and abilities to maintain a career, uphold finances, and pursue leisure activities. This knowledge is significant because many breast cancer patients stress that quality of life is just as important, if not more important, than quantity of life.

The effort that physicians take to inquire about QOL improves doctor-patient communication and shows patients that their physicians care about them and are interested in their well-being. To patients, this is highly meaningful.

### Future directions in the field of QOL assessments for breast cancer patients

#### Suggestions for the implementation of QOL assessments

Future implementation will not work by simply adding QOL measurements to the other daily tasks completed by the oncology staff. In order to provide timely response to patients' needs, QOL should be embedded into the care process of patient reported outcomes by redesigning care, particularly by paying attention to principles of effective distribution as well as implementing new infrastructures and technologies [18].

In order to have the ideal assessment system, it must be clinically relevant (useful and presented in a timely manner), sensitive to change, culturally sensitive, low burden, low in cost, built into the standard operating procedures, and meet regulatory, consumer, and community requirements [96]. QOL measures must be easily implemented into the medical office routine [93], requiring that they be short, easy to interpret, and not require intricate training or scoring [77,93]. These assessments need to be acceptable to the patients and be able to produce reports in real time without disrupting busy clinics [76]. Because of time constraints, patients, physicians, and office staff are not always willing to incorporate something new into their clinical routine [97]. Providing interpretations and recommendations about available resources and the score may be helpful rather than just providing the physician with a functional status score [77].

#### Suggested solutions to address challenges to implementation

While many healthcare professionals agree about the benefits of QOL assessments, few implement these measures because of the barriers presented. Table 1 provides suggested solutions to address the specific challenges to the implementation of QOL measurements in oncology practices.

**Table 1:** Suggested solutions to overcome the challenges of implementing QOL assessments into clinical practice

**Challenges**	**Solutions**
***Instrument characteristics***
QOL assessments provide "soft data."	Support the implementation of QOL assessments, as the soft data provides additional insights into a patient's health, as it provides qualitative data in addition to the quantitative data provided by "hard" measurement.
The types of questions asked can be too sensitive, personal, or irrelevant.	It is recommended that, when developing questionnaires, potential questions be tested by a population of elderly patients to gauge respondents' sensitivity and how effectively the questions measure a patient's QOL.
***Patient population***	
Questionnaires do not assess long-term survivors (over 5 years) – only 1 year survivors.	Long-term survivors of more than 5 years should be included in the original development and testing of instruments.
Among the elderly, there is illiteracy, worse compliance with questionnaires, and cognitive disorders.	The option of questionnaires administered in an interview format should be available to elderly patients. This solution would address compliance. In addition, it would address illiteracy and cognitive disorders, because the questionnaires could be read to the respondents, explained, and discussed with them.
***Healthcare professionals***	
Physicians are less familiar with how to utilize QOL assessments and how to interpret or respond to results.	Training classes about the importance, potential benefits, proper utilization of QOL assessments, interpretation of results, and appropriate action to be taken are recommended to be offered at medical schools and through Continuing Medical Education courses. To help better understand their utilization, healthcare providers could be taught whom the appropriate specialists to refer their patients would be based on the results of the QOL assessments.
Physicians do not have the proper tools needed to make QOL assessments part of their practice.	QOL assessments could be made accessible through online availability, allowing physicians to have a centralized location to download efficiently instruments as needed.
***Logistics and resources***	
Time limitations exist.	Questionnaires could be administered while the patient is waiting to be seen by the physician.
Measures are usually reported manually, which leads to inaccurate results and a long turnaround time.	The utilization of computerized assessments would improve the accuracy of QOL assessments and increase the efficiency of their use.
***Computerized assessment***	
Respondents may be unfamiliar with how to use computers or touchpad personal computers (PCs).	Brief training sessions of 10–15 minutes could be held while patients are in waiting rooms, where respondents would learn by the administrator how to manipulate the mouse, keyboard, and touchpad.
The programming of some questionnaires makes it difficult for patients to change their answers.	Efforts could be made to modify computer programming and software to facilitate computerized administration of questionnaires.

### The use of technology to facilitate the implementation of QOL assessments into clinical practice

Utilizing technology to implement QOL assessments into clinical practice has several advantages and makes it more feasible for physicians to use QOL information with their patients. Touch-screen computers facilitate use by patients, and those who have used this method favor it. Electronic methods of assessment are more accurate and less time-consuming than paper-and pencil questionnaires.

## Conclusion

In conclusion, we found that QOL assessment tools, including written assessments and computer assessments, have several advantages and are beneficial for breast cancer patients. The implementation of QOL assessments into clinical practice for breast cancer treatment has a high potential to benefit patients. Health-related quality of life has increasingly been an important factor to consider in the holistic treatment of breast cancer patients, and by providing accurate insights into QOL through self-reported questionnaires, physicians will be better able to make treatment decisions. Technologies can provide a highly efficient and accurate means of implementing QOL assessments so that they can help a wider range of breast cancer patients.

Physicians, nurses, and psychosocial staff recognize the merits of QOL assessments [26-28]. The advantages of implementing QOL tools are further supported by studies comparing intervention groups and controls, which have found that the intervention groups received more counseling and meaningful discussion with physicians. The information and advice gained in these counseling sessions have several benefits. In addition to learning about breast cancer, patients gain an understanding of how to best use the advice they received to improve the quality of their own lives. Because QOL instruments provide accurate assessments of the well-being and functionality of patients, utilizing them in clinical practice would significantly benefit patients and provide them with insights into their own care. Future studies involving the QOL of breast cancer patients should examine emerging science surround the implementation of QOL instruments, such as computerized versions and telephone-based applications, to enhance and streamline the assessment-taking process for both the patient as well as the medical staff. In order to create the most reliable and user-friendly application, potential users of the software should be included from the beginning to determine where possible problems might lie. Careful selection of the QOL assessment(s) should be considered with each population being examined to provide a reliable and valid patient-reported outcome.

## Supplementary Material

Additional file 1 Appendix A. Characteristics of generic and breast cancer-specific quality of life instruments (Instruments are listed alphabetically). Click here for file
