# Supplementary material for: Quality of life assessment in women with breast cancer: benefits, acceptability and utilization
Source: Health Qual Life Outcomes. 2007 May 2;5:24. doi: 10.1186/1477-7525-5-24 (PMC1877797; doi:10.1186/1477-7525-5-24)
Supplement: Additional file 1 — Appendix A. Characteristics of generic and breast cancer-specific quality of life instruments (Instruments are listed alphabetically). [file 1477-7525-5-24-S1.doc]

**Appendix A. Characteristics of generic and breast cancer-specific quality of life instruments (Instruments are listed alphabetically).**

|  | **Measure** | **Purpose** | **Domains** | **Scale** | **Time frame** | **No. of items** | **Administered by and**  **(completion time)** | **Reliability** | **Validity** |
| --- | --- | --- | --- | --- | --- | --- | --- | --- | --- |
| 1 | Beck Depression Inventory (BDI) [1] | Designed to measure depression | One domain:  Depression | Inventory produces a total score and is scaled on a four point Likert scale | Past two weeks | 21 | Self-administered  (5 minutes) | Reliabilities ranged from .48 to .86 | Correlation coefficients between the BDI and clinical ratings yield correlations from .55 to .96 [2] |
| 2 | Breast Cancer Chemotherapy Questionnaire  (BCQ) [3] | Developed to measure outcomes of women with stage II breast cancer receiving adjuvant chemotherapy | Seven domains:  Consequences of hair loss; emotional dysfunction; physical symptoms; trouble and inconvenience associated with treatment; fatigue; nausea; positive well-being | Seven point Likert scale ranging in responses | Past two weeks | 30 | Interviewer- administered  (10-15 minutes) | Internal consistency ranging from .89 to .91 | Correlation coefficients between BCQ and Spitzer QL-Index was .62 |
| 3 | Breast Cancer Prevention Trial Symptom Checklist  (BCPT) [4, 5] | Designed to examine the physical and psychological symptoms associated with menopause and Tamoxifen usage | Eight domains:  Hot flashes; nausea; bladder control; vaginal problems; musculoskeletal pain; cognitive problems; weight problems; arm problems | Five point Likert scale ranging from 0 (Not at all) to 4 (Extremely) | Past four weeks | 43 | Self-report  (estimated 30 minutes) | Reliability was .81 | Correlation coefficients between BCPT and SF-36 were -.40 and -.36 |
| 4 | Cancer Needs Questionnaire – Short Form  (CNQ-SF) [6] | Developed to assess cancer patients’ needs | Five domains: Psychological; health information; physical and daily living; patient care and support; interpersonal communication | Five point Likert scale ranging from 1 (No need/not applicable) to 5 (High need for help) | Unspecified | 32 | Self-administered  (estimated 15-20 minutes) | Reliability ranged from .77 to .94 | Correlation coefficients between CNQ-SF and EORTC QLQ-C30 and BDI ranged from .23 to .58 [7] |
| 5 | Cancer Rehabilitation Evaluation System  (CARES-SF) [8] | Developed to assess patients’ cancer-related problems | Six domains:  Physical; psychosocial; medical interaction; marital; sexual; global | Five point Likert scale ranging from 0 (Not at all) to 4 (Very much) | Past month | 59 | Self-administered  (average 20 minutes; Range of 10-34 minutes) | Reliabilities ranged from .39 to .82 | Correlation coefficients between the CARES-SF and the Memorial Symptom Assessment Scale (MSAS) ranged between .53 to .73 [9] |
| 6 | Center for Epidemiologic Studies Depression Scale-10  (CES-D) | Designed to measure depression | One domain:  Depression | Four point Likert scale ranging from 0 (Rarely) to 3 (Most of the time) and summed across the ten items to provide a total score. | Past week | 10 | Self-administered  (estimated 5 minutes) | Reliability of .92[10]; Reliability for CES-D 20 item scale is .86 [11] | Correctly identifies 98% of depressed patients as having major depression [10] |
| 7 | European Organization for Research and Treatment of Cancer QOL Breast Cancer Specific Version  (EORTC QLQ-BR23) [12] | Designed to measure QOL in the breast cancer population at various stages and with patients with differing modalities | Five domains:  Therapy side effects; arm symptoms; breast symptoms; body image; sexual functioning | Four point Likert scale ranging from 1 (Not at all) to 4 (Very much) | Past week | 23 | Self-report  (10 minutes) | Reliabilities ranged from .70 to .91 | Discriminant validity of mutually exclusive groups based on their initial performance status scores produced medium to large effect sizes ranging from .43 to 1.1 |
| 8 | European Organization for Research and Treatment of Cancer QOL Cancer Specific Version  (EORTC QLQ-C30) [13] | Cancer specific questionnaire designed to measure QOL in the cancer population | Nine domains:  Physical; role, cognitive; emotional; social; fatigue; pain; nausea and vomiting; global health status and quality of life | Four point Likert scale ranging from 1 (Not at all) to 4 (Very much); 1 (Very poor) to 7 (Excellent) | Past week | 30 | Self-administered  (Under 10 minutes) | Reliabilities ranged from .69 to .90.[14] Test-retest reliabilities ranged from .63 to .87 [15] | Correlation coefficient between the QLQ-C30 and the Profile of Mood States (POMS) was .56 [16]44]. |
| 9 | Edmonton Symptom Assessment System  (ESAS) [17] | Designed to measure a variety of symptoms | Nine domains:  Pain; tiredness; nausea; depression; anxiety; drowsiness; appetite; well-being; shortness of breath | Scaled using a visual analog scale | At the time of assessment | 9 | Self-administered  (estimated 5 minutes) | Internal consistency reliability of .39 to .86 | Correlation coefficient between the ESAS and the FACT was .85 [18] |
| 10 | Functional Assessment of Cancer Therapy – Breast Symptom Index (FACT-B) [19] | Specific to breast cancer patients | Six domains: Physical well-being; social/family well-being; emotional well-being; functional well-being; relationship with doctor; additional concerns | Five point Likert scale ranging from 0 (Not at all) to 4 (Very much) | Past week | 37 | Self-report or interviewer- administered  (estimated 25 minutes) | Internal consistency was .90 | Spearman correlations between FBSI and FACT ranged from .34 to .84 |
| 11 | Functional Assessment of Cancer Therapy – Endocrine System  (FACT-ES) [20] | Focus on endocrine concerns experienced during breast cancer treatment | One domain:  Endocrine concerns | Five point Likert scale ranging from 0 (Not at all) to 4 (Very much) and comprises a total score | Past week | 18 | Self-report or interviewer-administered  (estimated 10 minutes) | Internal consistency was .79  Test-retest reliability was .93 | Discriminant validity of known groups comparing adjuvant chemotherapy and those without any endocrine therapy produced a significant *t* score with the adjuvant chemotherapy group experiencing more endocrine symptoms than the non-endocrine therapy group |
| 12 | Functional Living Index – Cancer  (FLIC) [21] | Designed to assess the effect that cancer treatment and symptoms have on functional ability in all areas of life | Five domains:  Physical functioning; mental functioning; social functioning; general health/well-being; gastrointestinal symptoms | Seven point Likert-type linear analog scale. Patients are instructed to answer the questions by placing a vertical line at the point in the scale that best represents their response based on various Likert points along the scale | Past two weeks;  Past month;  Today | 22 | Self-administered  (Under 10 minutes) | Reliability ranged from .64 to .87.[21] | Correlation coefficients between FLIC and SF-36 ranged from .50 to .62 [22] |
| 13 | Geriatric Depression Scale – Short Form  (GDS-SF) [23] | Designed to assess depression in the elderly | Four domains:  Positive mood; sad mood; boredom, memory problems, and energy level; staying home | Scaled in a yes/no format | Past week | 15 | Self-administered  (estimated 5 minutes) | Internal consistency ranged from .60 to .77 [24] | Cut off score of greater than or equal to 7 in correctly diagnosing depression 79% of the time [25] |
| 14 | Hospital Anxiety and Depression Scale  (HADS) [26] | Developed to measure anxiety and depression | Two domains:  Anxiety and depression | Four point Likert scale | Past week | 14 | Self-administered  (estimated 5-10 minutes) | Reliabilities of .98 for total score, .85 for anxiety subscale, and .80 for depression subscale. Test-retest reliability has produced coefficients over a two month period for the total score, anxiety subscale, and depression subscale (.79, .79, and .63 respectively).[27] | Correlation coefficients between the HADS and Symptom Checklist 90 scale were .73 (anxiety subscale) and .67 (depression subscale [28]). |
| 15 | Life Satisfaction Questionnaire  (LSQ) [14] | Developed to measure one’s general sense of satisfaction with life as it relates to school, relationships, leisure time, religious practices, and overall health, specifically for women with breast cancer | Six domains:  Quality of family relation; physical symptoms; socioeconomic situation; quality of daily activities; sickness impact; and quality of close friend relation | Seven point Likert scale ranging from 1 (very much) to 7 (Not at all) | Past week | 32 | Self-report  (estimated 20 minutes) | Reliabilities ranged from .62 to .92 | Correlation coefficients between LSQ and EORTC QLQ-C30 were -.68 to .54 |
| 16 | Medical Outcome Short Form Health Survey  (SF-36) [29] | Developed to assess health-related QOL | Eight domains:  Physical functioning; role limitations due to physical health; role limitations due to emotional problems; energy/fatigue; emotional well-being; social functioning; bodily pain; general health | Scaled using various scales | Unspecified | 36 | Self-administered  (5 minutes) | Reliability ranged from .74 to .98 [30] | Correlation coefficients between the SF-36 and the General health Questionnaire (GHQ-29) were -.35 to =.61 (correlations are negative because the two scales run in opposite directions) [31] |
| 17 | Quality of Life Index  (QL-Index) [32] | Designed to assess health outcomes of those with cancer and other chronic diseases | Five domains:  Activity; daily living; health; support; outlook | Three point Likert Scale | Past two weeks | 5 | Interviewer-administered or self-administered  (Under 10 minutes) | Internal consistency of .78 | Correlation coefficients ranged from .40 to .63 .[32] |
| 18 | Rotterdam Symptoms Checklist – Modified [33] | Developed to assess symptom-related distress among cancer patients | Two domains:  Physical distress and miscellaneous variables | Four point Likert scale ranging from 1 (Not at all) to 4 (Very much) | Past week | 28 | Self-administered  (8 minutes) | Reliability of .88 | Correlation coefficients ranged from -.59 to -.61 when the Rotterdam Symptoms Checklist-Modified was compared with the SF-36 (correlations are negative because the two scales run in opposite directions) [33] |
| 19 | Satisfaction with Life Domains Scale for Breast Cancer  (SLDS-BC) [34] | Developed to measure satifaction with life among breast cancer patients | Five domains:  Social functioning; appearance; physical functioning; communication with medical providers; spirituality | Seven point Likert-type scale ranging from 1 (A “delighted” face) to 7 (A “very unhappy” face | Unspecified | 32 | Self-report  (estimated 20 minutes) | Reliabilities ranged from .90 to .93 | Correlation coefficient between SLDS-BC and FACT-B was .59 |
| 20 | World Health Organization Quality of Life – Brief Version  (WHOQOL-BREF) [35] | Designed to examine domain level profiles assessing quality of life | Four domains:  Physical health; psychological; social relationships; environment | Five point Likert scale with varying anchors | Past two weeks | 26 | Self-administered  (estimated 15-20 minutes) | Reliability ranged from .66 to .84. Similar alphas have been shown for test-retest reliability ranging from .66 to .87 | Correlation coefficients between the WHOQOL-BREF and SF-36 ranged from .36 to .78 [36] |
| 21 | Zung self-rating depression scale [37] | Designed to measure depression | Depression | Four point Likert scale ranging from 1 (A little of the time) to 4 (Most of the time) | Last 5 days | 20 | Self-administered  (estimated 10 minutes) | Internal consistency of .58[38] | Scale has produced good discriminant validity as it was found to be the primary discriminating variable in distinguishing depressed from nondepressed participants.  [39] |

**References:**
